# RETRACTION: Meta‐Analysis on the Efficacy of Traditional Chinese Medicine in Enhancing Surgical Site Wound Healing Post‐Colorectal Surgery

**DOI:** 10.1111/iwj.70564

**Published:** 2025-04-18

## Abstract

**RETRACTION:** LiuH.‐S. , QianH. , LiuJ. , and ZhangD. , “Meta‐Analysis on the Efficacy of Traditional Chinese Medicine in Enhancing Surgical Site Wound Healing Post‐Colorectal Surgery,” International Wound Journal 21, no. 3 (2024): e14444, 10.1111/iwj.14444.37953697 PMC10949938

The above article, published online on 12 November 2023, in Wiley Online Library (http://onlinelibrary.wiley.com/), has been retracted by agreement between the journal Editor in Chief, Professor Keith Harding; and John Wiley & Sons Ltd. Following an investigation by the publisher, all parties have concluded that this article was accepted solely on the basis of a compromised peer review process. The editors have therefore decided to retract the article. The corresponding author could not be reached for comment regarding the retraction.

**RETRACTION:** H.‐S. Liu , H. Qian , J. Liu , and D. Zhang , “Meta‐Analysis on the Efficacy of Traditional Chinese Medicine in Enhancing Surgical Site Wound Healing Post‐Colorectal Surgery,” International Wound Journal 21, no. 3 (2024): e14444, 10.1111/iwj.14444.37953697 PMC10949938

The above article, published online on 12 November 2023, in Wiley Online Library (http://onlinelibrary.wiley.com/), has been retracted by agreement between the journal Editor in Chief, Professor Keith Harding; and John Wiley & Sons Ltd. Following an investigation by the publisher, all parties have concluded that this article was accepted solely on the basis of a compromised peer review process. The editors have therefore decided to retract the article. The corresponding author could not be reached for comment regarding the retraction.

